# Supplementary material for: Oncologic and Long-Term Outcomes of Laparoscopic and Open Extended Cholecystectomy for Gallbladder Cancer
Source: J Clin Med. 2022 Apr 11;11(8):2132. doi: 10.3390/jcm11082132 (PMC9027168; doi:10.3390/jcm11082132)
Supplement: Supplementary file 1 [file jcm-11-02132-s001.zip › jcm-1646505-supplementary.pdf]

Supplementary Table S1. Univariable and Multivariable analyses of predictors of overall survival for 44 patients who underwent extended cholecystectomy.

	Univariate			Multivariate		
	**HR**	**95% CI**	***p*-Value**	**HR**	**95% CI**	***p*-Value**
**Age (years)**	1.064	1.013-1.117	0.014	1.465	1.151-1.865	0.002 *
**Male sex (vs female)**	1.095	0.387-2.15	0.831	92.516	3.97-2155.9	0.005 *
**Prothrombin time (INR)**	5.972	0.042-858.41	0.481			
**Preoperative bilirubin (mg/dL)**	1.965	0.944-4.093	0.071			
**Preoperative albumin, g/dL**	0.565	0.202-1.583	0.278			
**Preoperative CA19-9>37 U/mL**	9.490	2.053-43.878	0.004			
**Postoperative CA19-9 >37 U/mL**	7.641	2.571-22.712	<0.001	10.829	0.791-148.2	0.074
**Laparoscopic resection (vs open surgery)**	0.378	0.138-1.034	0.058			
**Liver resection type (ref: no liver resection)**			0.047			<0.001 *
Wedge	0.183	0.035-0.953	0.044	0.018	0-1.096	0.055
S4b/5	0.127	0.025-0.654	0.014	0.001	0-0.017	0.004 *
**Transfusion**	1.710	0.708-4.127	0.233	1193.8	15.451-92245.4	0.001 *
**LN metastasis**	5.086	1.995-12.965	0.001			
**T stage (T3 vs. T1,2)**	2.966	1.049-8.383	0.040	36.741	1.993-677.4	0.015 *
**Adjuvant chemotherapy**	1.005	0.418-2.414	0.992			

* Statistical significant *p*-value(<0.05).
